# The Red Queen and the persistence of linkage-disequilibrium oscillations in finite and infinite populations

**DOI:** 10.1186/1471-2148-7-211

**Published:** 2007-11-06

**Authors:** Roger D Kouyos, Marcel Salathé, Sebastian Bonhoeffer

**Affiliations:** 1 Institute of Integrative Biology, ETH Zürich, ETH-Zentrum CHN, 8092 Zürich, Switzerland

## Abstract

**Background:**

The Red Queen Hypothesis (RQH) suggests that the coevolutionary dynamics of host-parasite systems can generate selection for increased host recombination. Since host-parasite interactions often have a strong genetic basis, recombination between different hosts can increase the fraction of novel and potentially resistant offspring genotypes. A prerequisite for this mechanism is that host-parasite interactions generate persistent oscillations of linkage disequilibria (LD).

**Results:**

We use deterministic and stochastic models to investigate the persistence of LD oscillations and its impact on the RQH. The standard models of the Red Queen dynamics exhibit persistent LD oscillations under most circumstances. Here, we show that altering the standard model from discrete to continuous time or from simultaneous to sequential updating results in damped LD oscillations. This suggests that LD oscillations are structurally not robust. We then show that in a stochastic regime, drift can counteract this dampening and maintain the oscillations. In addition, we show that the amplitude of the oscillations and therefore the strength of the resulting selection for or against recombination are inversely proportional to the size of the (host) population.

**Conclusion:**

We find that host parasite-interactions cannot generally maintain oscillations in the absence of drift. As a consequence, the RQH can strongly depend on population size and should therefore not be interpreted as a purely deterministic hypothesis.

## Background

There is almost no species that is not attacked by parasites. From an evolutionary perspective, host-parasite interactions are of fundamental importance, because these interactions can have very strong fitness effects on both hosts and parasites. For any given species, it is hard to imagine a change of environment that is as fast, as consistent and as profound as that constituted by its coevolving parasite (or host) species. A particularly intriguing and much debated aspect of host-parasite coevolution is the so-called Red Queen Hypothesis (RQH) [1]. In a nutshell, the RQH states that the antagonistic coevolution between host and parasite leads to cyclical dynamics, also called Red Queen dynamics (RQD), which favor genetic shuffling [2-4]: The host continuously tries to escape the parasite which in turn responds to each adaptation with the corresponding counter-adaptation. In this arms race, recombination may offer the host the opportunity to generate novel, and therefore potentially resistant, offspring more quickly.

The only genetic effect of shuffling (through sex or recombination) is to reduce linkage disequilibria (LD), i.e. statistical associations between alleles at different loci in the genome. Thus, any theory that attempts to identify an evolutionary advantage of genetic shuffling must be based on a beneficial effect of reducing linkage disequilibria. In the context of the RQH, the benefit of genetic shuffling is associated with breaking up linkage disequilibria in the host population that are generated by selection pressures imposed by the coevolving parasite population. Specifically, a prerequisite for the RQH is that the host-parasite interaction generates persistent LD oscillations. If these LD oscillations would wane over time, then selection for recombination/sex can at best be transient. Indeed, standard models of the RQH [5-7] do show persistent LD oscillations. However, given that oscillations are often structurally not robust (structural robustness refers to whether the behavior of a system is robust to minor modifications of the form of the underlying equations), it is surprising that the question of robustness has been largely ignored in the context of the RQH.

Here, we investigate one of the standard models for the RQH [5], a discrete time model that uses a specific updating scheme where host and parasite frequencies are updated simultaneously. We then investigate two slightly modified models: (i) a continuous-time version of the standard model (referred to from here on as the continuous-time model), and (ii) a discrete-time version with a different updating scheme (referred to from here on as the sequential-updating model). While the standard model [5] includes most interaction types that have previously been studied (e.g. the matching allele [6,7] and the gene-for-gene interaction type [8,9]), we restrict our discussion to the matching-allele type (i.e. where the fitness of host and parasite depends only on the number of matched alleles) and neglect the (plant-specific) gene-for-gene type [8,9] which is generally known to exhibit selection against sex/recombination. In the first part of our analysis, we focus on the classical matching-allele type (considered in [5-7]) and then extend our scope to more general types of interaction [10].

Interestingly, we find that the proposed modifications of the standard model lead to a strong dampening of LD oscillations, which is driven by recombination and mutation. In addition, we find that even in the standard model, dampening occurs under some circumstances. These observations suggest that LD oscillations in the Red Queen are structurally not robust. If host-parasite interactions could maintain LD oscillations only transiently, the plausibility of the RQH would be severely undercut.

To investigate what factors could contribute to the persistence of LD oscillations we study here the effect of finite population size. In particular, we study the dynamic behavior of stochastic versions of both the standard simultaneous-updating model and of the alternative sequential-updating model. We have chosen to consider the sequential-updating model as a representative of the alternative models because it can be conveniently transformed into a stochastic model. We find that in the alternative model, drift indeed helps to maintain LD oscillations, and as a consequence, population size can strongly affect selection for recombination or sex. This contrasts the situation in the standard model where the effects of drift are much less pronounced and restricted to weak selection coefficients. Finally, we consider the evolution of sex/recombination in a broader set of interaction types (including the matching-allele type) and show that our results hold for the majority of these interaction types.

## Results

### The structural robustness of LD oscillations in deterministic models

**Figure 1:**
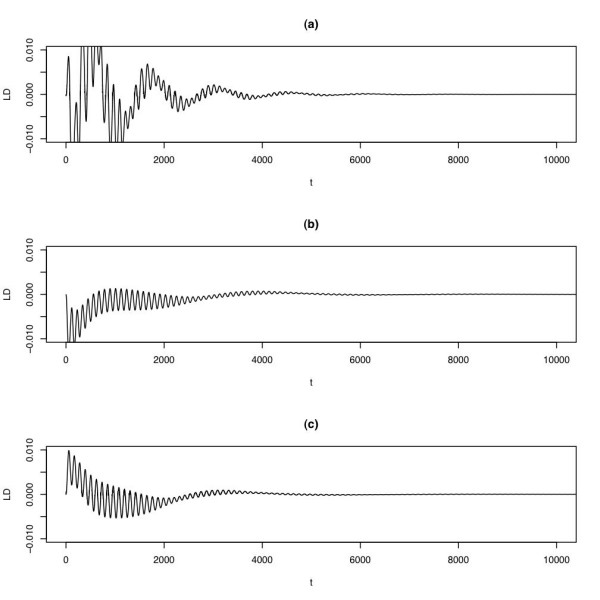
Typical simulation run in the deterministic versions of the standard model **(a)**, the sequential-updating model **(b)**, and the continuous-time model **(c)**. The variable *t *represents the number of host generations. The figures show the LD oscillations during 10000 host generations. The following parameters were used: *r*_*mm *_= 0.1, *s*_*H *_= *s*_*P *_= 0.05.

A peculiar and hitherto unnoticed consequence of deterministic modeling of host-parasite coevolution is that the amplitude of the resulting linkage disequilibrium (LD) oscillations may decrease over time and eventually reach negligibly small values. LD oscillations can be damped in all three models (see Figure 1 for typical simulation runs). The dampening is a general phenomenon in the two alternative models, but in the standard model, it is only observed when selection coefficients are small (approximately *s*_*H *_and *s*_*P*_*<*0.4; see section "Methods"). Moreover, for weak selection, the type of LD oscillations in the standard model depends on the initial conditions: either the oscillations are weak but stable, or the oscillations are damped. For random initial allele frequencies, both scenarios seem to occur with similar frequency.

Previous analyses of the standard model [5-7] did not report any dampening. The dampening of the LD oscillations may have gone unnoticed for several reasons. First, for very weak selection, the dampening is particularly slow. Second, in the standard model (but not in the alternative models), only the LD oscillations are damped while the genotype-frequencies remain oscillating with a stable amplitude (results not shown). Third, the dampening does not occur for strong selection, even if selection is strong only for the parasite (this is the parameter range analyzed in [6,7]). Overall, our simulations show that stability of LD oscillations is not a generic feature of the standard model, unless selection is strong on the parasite.

The sequential updating model exhibits substantial dampening of the LD oscillations for various recombination rates, mutation rates and selection coefficients ranging over several orders of magnitude (see supplementary table S1; all supplementary figures and tables can be found in the Additional file 1). Moreover, the rate of the dampening depends on both the mutation rate and the recombination rate (see supplementary table S1, Additional file 1), with high mutation and recombination rates causing faster decay. The continuous-time model shows a qualitatively similar behavior (results not shown). These patterns suggest that for the alternative models, the antagonistic interactions between hosts and parasites alone are not able to maintain genotype- and LD oscillations against the dampening forces of mutation and recombination. Taken together, these results suggest that stable LD oscillations are not a robust feature of RQ dynamics.

### Testing the impact of stochasticity

In order to analyze the effects of (finite) population size on the RQH, we developed stochastic versions of our models (for the sake of simplicity we considered stochastic effects only for the host populations). This is straightforward for the discrete-time models (see section "Methods"), but more cumbersome for continuous time models. We therefore focus on the two discrete-time models: The standard model illustrates the role of stochasticity for systems in which oscillations can be maintained deterministically, and the alternative (sequential-updating) model illustrates its role for systems in which oscillations are damped deterministically.

#### Stochasticity can help to maintain the LD oscillations

**Figure 2:**
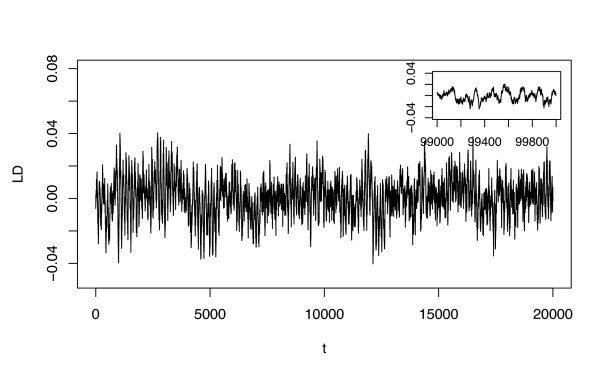
Typical simulation run in the stochastic version (*N *= 10 000) of the sequential-updating model. The variable t represents host generations. The main figure shows the LD oscillations from the start, the inset shows the dynamics of the LD oscillations later during the simulation (*t *= 100 000). The following parameters were used: *r*_*mm *_= 0.1, *s*_*H *_= *s*_*P *_= 0.05.

**Figure 3:**
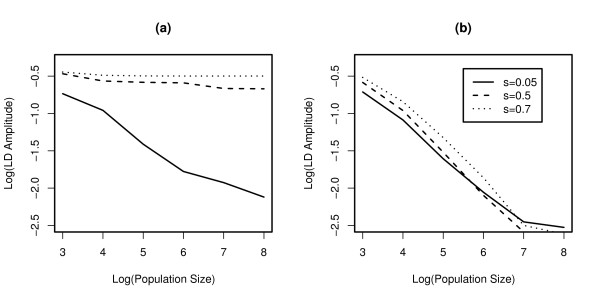
The effect of population size *N *on the amplitude of LD oscillations in the standard model **(a) **and in the sequential-updating model **(b)**. The amplitude of the LD oscillations is measured as the maximum difference of linkage disequilibrium (Δ*LD*) between t = 10 000 and t = 20 000. The following parameter was used: *r*_*mm *_= 0.1.

In the stochastic regime, LD oscillations can be maintained indefinitely even in the alternative model (Figure 2). This result holds for a wide range of mutation rates, recombination rates, and population sizes (see supplementary table S1, Additional file 1). Of special interest is the impact of population size on the LD amplitude that can be maintained at steady state (shown in Figure 3 for both the standard and the alternative model). In the standard model, stochasticity increases the amplitudes of LD oscillations only for small selection coefficients, i.e. for those parameter values where damped oscillations occur in the deterministic model. In the alternative model, on the other hand, the amplitudes of LD oscillations increase with decreasing population size independent of selection coefficients. In particular, the amplitudes of LD oscillations tend to negligibly small values as population size grows to very large values (i.e. as the system approaches the deterministic range). Thus, while mutation and recombination can dampen the LD oscillations, finite population size counters this process.

#### Consequences for the evolution of recombination

How does the dampening of the LD oscillations affect the evolution of sex and recombination? The mechanism underlying the RQH requires fluctuations of LD and epistasis (note that epistasis in hosts reflects LD in parasites and vice versa), which in turn strongly depend on population size (Figure 3). Thus, an effect of population size on the evolution of recombination seems evident.

To analyze this effect, we distinguish between two cases: (i) the competition between a sexual and a completely asexual type and (ii) the competition between two sexual types with different recombination rates. Both cases can be considered with the same model (and are in fact just two cases among a continuum of scenarios given by different recombination rates between the selected loci and between the modifier and the selected loci) but correspond to two different biological scenarios: The first case describes the replacement of a sexual by an asexual population. The second case describes the successive change of recombination rate. Several studies [5-7] have shown that the RQH depends crucially on the linkage between the modifier and the genes involved in host-parasite interactions. In particular, selection for higher recombination rates is generally more favored when linkage is tight. In the competition between sexual and asexual types, recombination occurs only between sexuals (because the asexuals reproduce clonally). Therefore, the modifier responsible for asexuality never switches its genetic background, i.e. it is tightly linked to the rest of the genome. Thus, the requirements that need to be met for the RQD to favor sexual over asexual types are less stringent than those that need to be met to favor high over low recombination rates, since in the latter case, the modifier can recombine away from the genotypic combinations that it produces (note, however, that the conditions are less stringent only if there is no ---or very little--- cost to sex). We model the competition between sexuals and asexuals by setting the recombination rates *r*_*mm *_and *r*_*mM *_to 0, and *r*_*MM*_*> *0 (see section "Methods") and the competition between modifiers coding for low and high recombination rate by choosing recombination rates 0 <*r*_*mm *_<*r*_*mM *_< r_*MM*._. Note that the modifier is selectively neutral, i.e. costs of sex are not included in our simulations.

**Figure 4:**
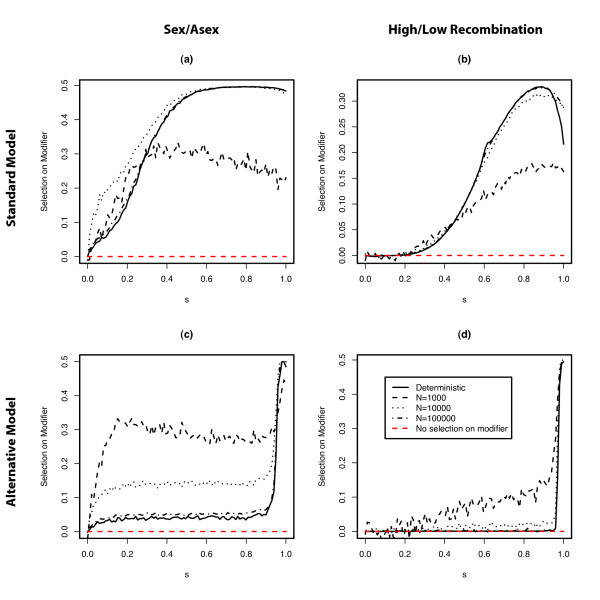
Selective advantage of sexual over asexual reproduction (*r*_*mm *_= 0, *r*_*mM *_= 0, *r*_*MM *_= 0.1) **(a,c) **and of high over low recombination **(b,d) **in both the standard model **(a,b) **and the sequential-updating model **(c,d)**, plotted for different selection coefficients (*s*) and different population sizes. The y-axis corresponds to the selection on the modifier (of sex/recombination), which is measured as the frequency change during a simulation run (i.e. 1000 host generations after introduction of the modifier allele M), averaged over 10000 simulation runs (100 for the deterministic model) with different random initial conditions.

Figure 4 shows the impact of population size on the evolution of recombination for both the standard model (panels a and b) and for the alternative model (panel c and d). Notice that we measure the selection on the modifier between generations 1000 and 2000 (see section methods). Thus, although the LD oscillations (and with them the selection on the modifier) vanish eventually in the cases with damped oscillations, they are still present (although very weakly) when we track the modifier. In the standard model, drift increases the strength of selection on the modifier if selection on the interaction loci is weak but decreases the strength of selection on the modifier when selection on the interaction loci is strong. In the first region, damped LD oscillations occur and drift considerably increases the amplitude of the oscillations, whereas the LD oscillations are always stable in the second region and drift affects the amplitude only marginally (see also Figure 3). This pattern suggests that drift increases the strength of selection on the modifier if it increases the amplitude of the LD oscillations, but rather decreases this selection if it does not affect the LD oscillations. In accordance with this interpretation, drift always increases the strength of selection on the modifier in the alternative model (in which LD oscillations are always damped), i.e. the strength of selection on the modifier strongly increases with decreasing population size.

#### The impact of the interaction type

**Figure 5:**
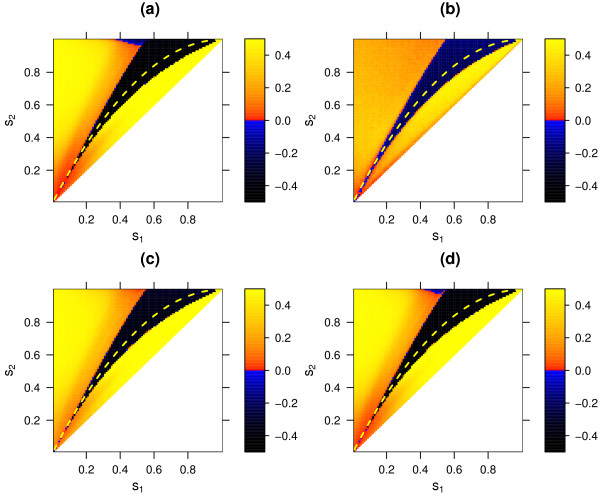
Selective advantage of sexual over asexual reproduction (*r*_*mm *_= 0, *r*_*mM *_= 0, *r*_*MM *_= 0.1) in the standard model for different interaction matrices, simulated deterministically **(a) **and stochastically with populations sizes *N *= 1000 **(b)**, *N *= 10 000 **(c)**, and *N *= 100 000 **(d)**. The color of each of the points on a graph shows the frequency change during a simulation run (i.e. 1000 host generations after introduction of the modifier allele M), averaged over 10000 simulation runs (100 for the deterministic model) with different random initial conditions. The number of host alleles, *n*, matched by the parasite determines the corresponding fitness *w*_*n *_in the following way: for hosts, *w*_2 _= 1-*s*_2_*, w*_1 _= 1 *- s*_1_*, w*_0 _= 1, for parasites *w*_2 _= 1, *w*_1 _= (*1 *- *s*_2_)/(1 - *s*_1_), *w*_0 _= 1 - *s*_2_. This generalized model reflects the biological characteristic that host and parasite fitness are inversely correlated. The matrix can be continually varied as a function of the parameters *s*_1 _and *s*_2_, and contains the MA and the MMA as special cases (MA: *s*_1 _= 0; MMA, *s*_2 _= 1-(*1*-*s*_1_)^2^, yellow dashed line). Only fitness matrices with *s*_1 _≦ *s*_2 _are shown. The parameters *s*_1 _and *s*_2 _range from 0 to 1 with 0.01 gradation.

**Figure 6:**
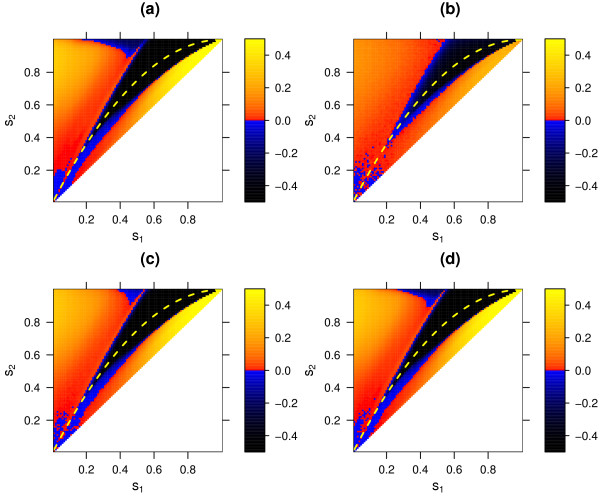
Selective advantage of a modifier increasing the recombination rate in the standard model(*r*_*mm *_= 0.1, *r*_*mM *_= 0.15, *r*_*MM *_= 0.2), for the deterministic regime **(a) **and for the stochastic regime with population size *N *= 1000 **(b)**, *N *= 10 000 **(c)**, and *N *= 100 000 **(d)**. For further details see legend of figure 5.

**Figure 7:**
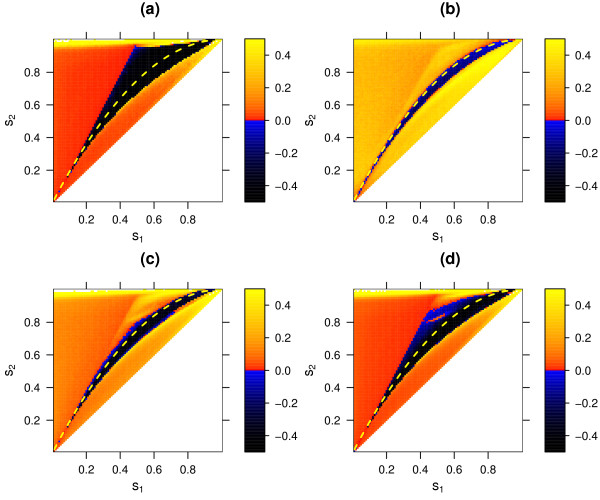
Selective advantage of sexual over asexual reproduction (*r*_*mm *_= 0, *r*_*mM *_= 0, *r*_*MM *_= 0.1) in the sequential-updating model for different interaction matrices, simulated deterministically **(a) **and stochastically with populations sizes *N *= 1000 **(b)**, *N *= 10 000 **(c)**, and *N *= 100 000 **(d)**. For further details see legend of figure 5.

**Figure 8:**
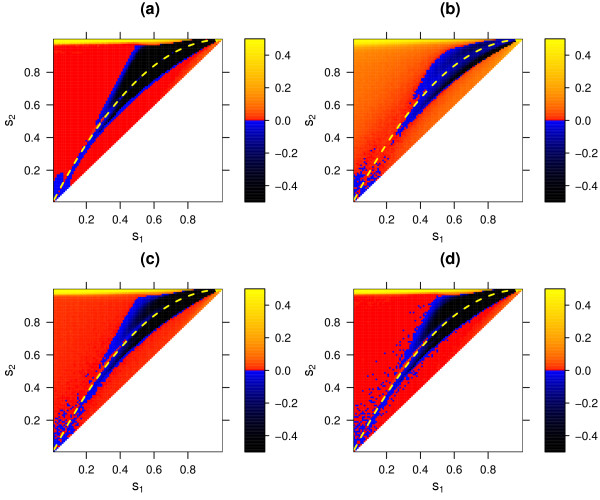
Selective advantage of a modifier increasing the recombination rate in the sequential-updating model (*r*_*mm *_= 0.1, *r*_*mM *_= 0.15, *r*_*MM *_= 0.2), for the deterministic regime **(a) **and for the stochastic regime with population size *N *= 1000 **(b)**, *N *= 10 000 **(c)**, and *N *= 100 000 **(d)**. For further details see legend of figure 5.

The preceding discussion was based on the matching allele interaction type. In order to investigate the impact of the interaction type, we measured the selection on a recombination/sex modifier for the generalized matching allele interaction type (see section "Methods") with selection coefficients *s*_1 _and *s*_2 _ranging from 0 to 1 with a gradation of 0.01 (i.e. for 10^4 ^different interaction matrices). The results are summarized in Figures 5, 6, 7, 8. Figures 5 (selection for/against sex) and 6 (selection for lower/higher recombination) correspond to the standard model and Figures 7 and 8 to the alternative model. These figures show that for most interaction types, the impact of population size is qualitatively the same as for the matching allele interaction type: In the standard model, drift only increases the strength of selection on the modifier when *s*_1 _and *s*_2 _are small. In the alternative model, however, drift increases the strength of selection for the whole range of *s*_1 _and *s*_2_. The only exception to this pattern can be found in the in the vicinity of the MMA (i.e when the number of matched loci determines the fitness in an approximately multiplicative way). In this region, we observe a strong selection against sex/recombination even in the deterministic regime. The figures show that with decreasing population size this region (where selection against recombination is strong) shrinks and the strength of selection against sex/recombination decreases. The reason for this effect is that systems with an (almost) multiplicative fitness function exhibit a different dynamic behavior than those with stronger interactions: Instead of periodic alternations of positive and negative LD, the system converges to a state in which the LD does not change its sign anymore (see discussion). Other than this effect around the MMA, finite population size does not have any appreciable effect on the direction of selection on the modifier: Independently of population size, sex wins against asex for the vast majority of interaction types (in both the standard and alternative model), and high recombination wins against low recombination only if the selection coefficients are strong enough.

## Discussion

Our results show that damped LD oscillations are the rule for the sequential-updating and the continuous-time model. In the standard model however, damped LD oscillations occur rarely (i.e. only when selection is weak). Moreover, persistent oscillations have also been observed in a model with interaction types different from the ones used here [11,12]. Thus, the dampening of the LD oscillations depends on the specific implementation of the host-parasite interaction. This raises the question whether damped or persistent oscillations are the generic case. Which of the models investigated here is most applicable to any particular host-parasite interaction strongly depends on the corresponding biology. The sequential-updating model, for example, describes a situation in which the parasite population can first adapt to the host population for a few generations, and only subsequently affects host fitness. This pattern might be realized if at the start of every host-generation, the parasite population is small (and hence exerts only a negligible selection on the host-population) and later, after a number of parasite generations, reaches an appreciable size (and thus exerts appreciable selection on the host after it has adapted to the host population). The standard model assumes that the fitness-effect of a host-parasite interaction affects host and parasites simultaneously. Which scenario is more plausible is, in our view, debatable. More important, however, is the fact that oscillations are also damped in the continuous time model (in which updating is by definition simultaneous), suggesting that (i) the dampening is not an artifact that is particular only to the sequential updating model and (ii) that the dampening cannot be attributed exclusively to sequential versus simultaneous updating. The difference in the dynamical behavior of the standard model and the continuous-time model is in line with the general observation that discrete-time models tend to show more oscillatory behavior [13]. Continuous-time models are commonly considered more appropriate when reproduction is not fully synchronous. Finally, the view that persistent LD oscillations are structurally not robust is further supported by the fact that even the standard model exhibits damped-oscillations when selection is weak.

The observation that the persistence of LD oscillations may not be structurally robust places a larger emphasis on the possible role of stochastic effects in the context of the RQH. If oscillations are persistent in the deterministic model, then drift is not required to maintain considerable oscillations and population size only has a modest impact (Figure 4). If, however, the oscillations are damped in the deterministic model, then drift is a necessary requirement for persistent RQ dynamics and the strength of the selection for recombination depends crucially on the population size. In this latter case, the RQH should be considered as an essentially stochastic hypothesis, in contrast to the traditional view which classifies it as deterministic [14].

In the alternative model (and in the standard model with small selection), we generally observe stronger selection on modifiers of sex and recombination in small populations. This could be explained by two main mechanisms. The first mechanism is based on the observation that the amplitude of the LD- and epistasis- oscillations depend strongly on the population size (see Figure 3), reaching the largest values for small populations. This explains the strong selection in small populations (because the effect of recombination directly reflects the strength of LD). Alternatively, the impact of population size could merely reflect a superposition of the RQH (as described in the deterministic model) and the well-known Fisher-Muller effect, which states that in finite population under directional selection, recombination can increase the response to selection [15]. However, we found that if the corresponding deterministic model exhibits persistent oscillations, decreasing population size does not increase the strength of selection on the modifier (e.g. in the standard model with strong selection; see Figure 4). This speaks against the Fisher-Muller effect but is in line with the expectation based on the first mechanism. We therefore think that the first mechanism is more plausible, although it is conceivable that the Fisher-Muller effect also contributes to selection on the recombination modifier.

On first thought, the observation that sex/recombination is strongly selected against for interaction types close to the MMA seems paradoxical, because both host and parasite loci determine fitness in a multiplicative fashion. Thus, one might expect that there is no epistasis. As a consequence, no LD should build up, and there should be no selection for or against sex in the region close to the MMA ("black crescent" in Figure 5a). This expectation, however, does not take into account that the epistasis experienced by the host population is a result of both the interaction matrix (i.e. *s*_1 _and *s*_2_) and the disequilibrium of the parasite population (see expression for host fitness in section "model"). In our simulations, we see that if recombination is small, host and parasites that interact according to the MMA (or closely related interaction types) seem to behave in a very particular way: If the system starts exactly at the "central equilibrium" (all frequencies are equal to 1/4 and thus LD equals 0), the system stays there and no LD develops. If, however, a small LD is introduced, the system leaves the central equilibrium and approaches a stable state with a strong and constant LD, which is either positive or negative depending on the initial condition. An example for this behavior is given in supplementary figure S3, Additional file 1. In accordance with the reduction principle [16], such a steady state with non-vanishing LD leads to selection against recombination and thus explains our results for the MMA and the closely related interaction types. Interestingly, a similar behavior has been described in diploid single-species models where a population can attain high-complementarity equilibria (HCE), i.e. two stable steady states with non-vanishing LDs of opposite sign [17].

## Conclusion

We conclude that in deterministic models of host-parasite interactions, persistent LD oscillations are structurally not robust. Whether these oscillations are persistent or damped depends on both the implementation of the model and on the parameter region. In this article, we found damped LD oscillations for weak selection in one of the standard models for host-parasite interactions [5] and throughout the whole parameter range (in which oscillations existed at all) in two alternative versions of this model. Damped LD oscillations imply (i) only transient selection for or against sex/recombination in the deterministic regime, and (ii) a large impact of stochasticity (i.e. of finite populations) on the evolution of sex/recombination. If oscillations are damped in the deterministic regime, stochasticity can counteract the dampening and maintain LD oscillations with considerable amplitude, which becomes larger with smaller population size. As a consequence, selection for or against sex/recombination is persistent in the stochastic regime and depends inversely on population size. If, on the other hand, LD oscillations are persistent in the deterministic regime, then stochasticity only weakly affects the evolution of sex/recombination and rather decreases the selection on a modifier. Overall, the fact that LD-oscillations are structurally not robust in deterministic models of the RQH and that consequently, the impact of host-parasite interactions may crucially depend on population size, suggests that the RQH should be seen as an essentially stochastic hypothesis.

## Methods

### The deterministic model

Both hosts and parasites have 2 interaction loci with two alternative alleles (0 and 1) that determine the interaction between hosts and parasite. Additionally, the host has a modifier locus with two possible alleles, m (wildtype) and M (mutant), that affect the recombination rate. Hence, the eight possible host genotypes are 00m, 00M, 01m, 01M, 10m, 10M, 11m and 11M. The host population is initiated with random genotype frequencies, except that initially all hosts have the wildtype allele m at the modifier locus. The parasite population consists of four genotypes (00,01,10,11) and is also initiated with random genotype frequencies.

In the discrete-time models (the standard model and the sequential-updating model), the following processes occur at each timestep: host reproduction, host selection, host mutation, pathogen reproduction, pathogen selection, and pathogen mutation. The difference between the two discrete-time models is that in the standard model, host and parasite populations are updated simultaneously, whereas in the alternative model, they are updated sequentially. To be more explicit, the updating in the sequential model works as follows. We first let the parasite population interact with the current host population, and then update the parasite population accordingly. This step is iterated *n*_*pg *_times, where *n*_*pg *_denotes the number of pathogen generations per host generation (for all simulations, we use *n*_*pg *_= *5*). Then we update the host population by letting it interact with the updated parasite population. In the standard model, on the other hand, both populations are updated simultaneously after one round of interaction. In this model, different generation times in host and parasites are realized by replacing every generation the whole parasite population but only a fraction 1/*n*_*pg *_of the host population. The equations of the two discrete-time models read as follows:

fP(t+1)=RMSp([fH(t);fP(t)])fH(t+1)=(1−1npg)fH(t)+(1npg)RMSh([fH(t);fP(t)])
 MathType@MTEF@5@5@+=feaafiart1ev1aaatCvAUfKttLearuWrP9MDH5MBPbIqV92AaeXatLxBI9gBaebbnrfifHhDYfgasaacPC6xNi=xI8qiVKYPFjYdHaVhbbf9v8qqaqFr0xc9vqFj0dXdbba91qpepeI8k8fiI+fsY=rqGqVepae9pg0db9vqaiVgFr0xfr=xfr=xc9adbaqaaeGacaGaaiaabeqaaeqabiWaaaGcbaqbaeqabiqaaaqaaiabdAgaMnaaCaaaleqabaGaemiuaafaaOGaeiikaGIaemiDaqNaey4kaSIaeGymaeJaeiykaKIaeyypa0JaemOuaiLaemyta0Kaem4uam1aaSbaaSqaaiabdchaWbqabaGccqGGOaakcqGGBbWwcqWGMbGzdaahaaWcbeqaaiabdIeaibaakiabcIcaOiabdsha0jabcMcaPiabcUda7iabdAgaMnaaCaaaleqabaGaemiuaafaaOGaeiikaGIaemiDaqNaeiykaKIaeiyxa0LaeiykaKcabaGaemOzay2aaWbaaSqabeaacqWGibasaaGccqGGOaakcqWG0baDcqGHRaWkcqaIXaqmcqGGPaqkcqGH9aqpdaqadaqaaiabigdaXiabgkHiTKqbaoaalaaabaGaeGymaedabaGaemOBa42aaSbaaeaacqWGWbaCcqWGNbWzaeqaaaaaaOGaayjkaiaawMcaaiabdAgaMnaaCaaaleqabaGaemisaGeaaOGaeiikaGIaemiDaqNaeiykaKIaey4kaSYaaeWaaeaajuaGdaWcaaqaaiabigdaXaqaaiabd6gaUnaaBaaabaGaemiCaaNaem4zaCgabeaaaaaakiaawIcacaGLPaaacqWGsbGucqWGnbqtcqWGtbWudaWgaaWcbaGaemiAaGgabeaakiabcIcaOiabcUfaBjabdAgaMnaaCaaaleqabaGaemisaGeaaOGaeiikaGIaemiDaqNaeiykaKIaei4oaSJaemOzay2aaWbaaSqabeaacqWGqbauaaGccqGGOaakcqWG0baDcqGGPaqkcqGGDbqxcqGGPaqkaaaaaa@809F@

for the standard model, and

fP(t+1)=RMSp(npg)([fH(t);fP(t)])fH(t+1)=RMSh([fH(t);fP(t+1)]
 MathType@MTEF@5@5@+=feaafiart1ev1aaatCvAUfKttLearuWrP9MDH5MBPbIqV92AaeXatLxBI9gBaebbnrfifHhDYfgasaacPC6xNi=xI8qiVKYPFjYdHaVhbbf9v8qqaqFr0xc9vqFj0dXdbba91qpepeI8k8fiI+fsY=rqGqVepae9pg0db9vqaiVgFr0xfr=xfr=xc9adbaqaaeGacaGaaiaabeqaaeqabiWaaaGcbaqbaeqabiqaaaqaaiabdAgaMnaaCaaaleqabaGaemiuaafaaOGaeiikaGIaemiDaqNaey4kaSIaeGymaeJaeiykaKIaeyypa0JaemOuaiLaemyta0Kaem4uam1aa0baaSqaaiabdchaWbqaaiabcIcaOiabd6gaUnaaBaaameaacqWGWbaCcqWGNbWzaeqaaSGaeiykaKcaaOGaeiikaGIaei4waSLaemOzay2aaWbaaSqabeaacqWGibasaaGccqGGOaakcqWG0baDcqGGPaqkcqGG7aWocqWGMbGzdaahaaWcbeqaaiabdcfaqbaakiabcIcaOiabdsha0jabcMcaPiabc2faDjabcMcaPaqaaiabdAgaMnaaCaaaleqabaGaemisaGeaaOGaeiikaGIaemiDaqNaey4kaSIaeGymaeJaeiykaKIaeyypa0JaemOuaiLaemyta0Kaem4uam1aaSbaaSqaaiabdIgaObqabaGccqGGOaakcqGGBbWwcqWGMbGzdaahaaWcbeqaaiabdIeaibaakiabcIcaOiabdsha0jabcMcaPiabcUda7iabdAgaMnaaCaaaleqabaGaemiuaafaaOGaeiikaGIaemiDaqNaey4kaSIaeGymaeJaeiykaKIaeiyxa0faaaaa@7052@

for the sequential-updating model. The functions *RMS*_*h *_and *RMS*_*p *_denote the successive action of selection, mutation and recombination on the genotype frequencies (*f*^*H *^and *f*^*P*^)for hosts and parasites respectively. The superscript (*n*_*pg*_) expresses that corresponding function is iterated *n*_*pg *_times.

The continuous-time model used here can be obtained from the standard model by replacing a fraction Δ*t *of the host population and a fraction *n*_*pg*_Δ*t *of the parasite population every time step and then taking the limit Δ*t*→0. The resulting ODEs read

dfP(t)dt=(RMSp([fH(t);fP(t)])−fP(t))dfH(t)dt=1npg(RMSh([fH(t);fP(t)])−fH(t)).
 MathType@MTEF@5@5@+=feaafiart1ev1aaatCvAUfKttLearuWrP9MDH5MBPbIqV92AaeXatLxBI9gBaebbnrfifHhDYfgasaacPC6xNi=xI8qiVKYPFjYdHaVhbbf9v8qqaqFr0xc9vqFj0dXdbba91qpepeI8k8fiI+fsY=rqGqVepae9pg0db9vqaiVgFr0xfr=xfr=xc9adbaqaaeGacaGaaiaabeqaaeqabiWaaaGcbaqcfaybaeqabiqaaaqaamaalaaabaGaemizaqMaemOzay2aaWbaaeqabaGaemiuaafaaiabcIcaOiabdsha0jabcMcaPaqaaiabdsgaKjabdsha0baacqGH9aqpcqGGOaakcqWGsbGucqWGnbqtcqWGtbWudaWgaaqaaiabdchaWbqabaGaeiikaGIaei4waSLaemOzay2aaWbaaeqabaGaemisaGeaaiabcIcaOiabdsha0jabcMcaPiabcUda7iabdAgaMnaaCaaabeqaaiabdcfaqbaacqGGOaakcqWG0baDcqGGPaqkcqGGDbqxcqGGPaqkcqGHsislcqWGMbGzdaahaaqabeaacqWGqbauaaGaeiikaGIaemiDaqNaeiykaKIaeiykaKcabaWaaSaaaeaacqWGKbazcqWGMbGzdaahaaqabeaacqWGibasaaGaeiikaGIaemiDaqNaeiykaKcabaGaemizaqMaemiDaqhaaiabg2da9maalaaabaGaeGymaedabaGaemOBa42aaSbaaeaacqWGWbaCcqWGNbWzaeqaaaaacqGGOaakcqWGsbGucqWGnbqtcqWGtbWudaWgaaqaaiabdIgaObqabaGaeiikaGIaei4waSLaemOzay2aaWbaaeqabaGaemisaGeaaiabcIcaOiabdsha0jabcMcaPiabcUda7iabdAgaMnaaCaaabeqaaiabdcfaqbaacqGGOaakcqWG0baDcqGGPaqkcqGGDbqxcqGGPaqkcqGHsislcqWGMbGzdaahaaqabeaacqWGibasaaGaeiikaGIaemiDaqNaeiykaKIaeiykaKcaaiabc6caUaaa@84B0@

In all models, hosts reproduce by recombining their genotypes, while parasites reproduce clonally. Recombination of two host genomes occurs at a rate that depends on the allele at the modifier locus in the two recombining hosts. If both hosts have allele *m*, the (per genome) recombination rate is *r*_*mm *_(i.e. the wildtype recombination rate). If one host has allele *m *and the other has allele *M*, the recombination rate is *r*_*mM*_. If both hosts have allele *M*, the recombination rate is *r*_*MM*_. We allow a maximum of one recombination event per genome, and the probability of the crossing-over event is independent of its location on the genome. Selection is determined by the fitness matrices *w*^*H*^_*ij *_and *w*^*P*^_*ij*_. Specifically, *w*^*H*^_*ij *_denotes the fitness of a host-genotype *i *interacting with a parasite-genotype *j *and *w*^*P*^_*ij *_denotes the fitness of the parasite-genotype *i *interacting with a host genotype *j*. Since the interaction probability for host *i *and parasite *j *is proportional to their frequencies, *f*^*H*^_*i *_and *f*^*P*^_*j*_, the fitness of the host-genotype i reads

wiH=∑jwijHfjP
 MathType@MTEF@5@5@+=feaafiart1ev1aaatCvAUfKttLearuWrP9MDH5MBPbIqV92AaeXatLxBI9gBaebbnrfifHhDYfgasaacPC6xNi=xI8qiVKYPFjYdHaVhbbf9v8qqaqFr0xc9vqFj0dXdbba91qpepeI8k8fiI+fsY=rqGqVepae9pg0db9vqaiVgFr0xfr=xfr=xc9adbaqaaeGacaGaaiaabeqaaeqabiWaaaGcbaGaem4DaC3aa0baaSqaaiabdMgaPbqaaiabdIeaibaakiabg2da9maaqafabaGaem4DaC3aa0baaSqaaiabdMgaPjabdQgaQbqaaiabdIeaibaaaeaacqWGQbGAaeqaniabggHiLdGccqWGMbGzdaqhaaWcbaGaemOAaOgabaGaemiuaafaaaaa@3E45@

and thus the frequency of the host-genotype i after selection is

f'iH=fiHwiH∑kwkHfkH
 MathType@MTEF@5@5@+=feaafiart1ev1aaatCvAUfKttLearuWrP9MDH5MBPbIqV92AaeXatLxBI9gBaebbnrfifHhDYfgasaacPC6xNi=xI8qiVKYPFjYdHaVhbbf9v8qqaqFr0xc9vqFj0dXdbba91qpepeI8k8fiI+fsY=rqGqVepae9pg0db9vqaiVgFr0xfr=xfr=xc9adbaqaaeGacaGaaiaabeqaaeqabiWaaaGcbaGaemOzayMaei4jaCYaa0baaSqaaiabdMgaPbqaaiabdIeaibaakiabg2da9iabdAgaMnaaDaaaleaacqWGPbqAaeaacqWGibasaaqcfa4aaSaaaeaacqWG3bWDdaqhaaqaaiabdMgaPbqaaiabdIeaibaaaeaadaaeqbqaaiabdEha3naaDaaabaGaem4AaSgabaGaemisaGeaaiabdAgaMnaaDaaabaGaem4AaSgabaGaemisaGeaaaqaaiabdUgaRbqabiabggHiLdaaaaaa@4608@

The parasite frequencies after selection are calculated analogously. Each interaction locus mutates independently with probability *μ *= *10*^-5 ^per generation, and forward and backward mutations occur with the same probability.

### The stochastic model

We turn the deterministic into a stochastic model with population size *N *by including a sampling step each generation (after the mutation step): *N *individuals are sampled according to the multinomial distribution with frequencies *f*^*H*^_*i*_. The genotype frequencies are then obtained by dividing the number of sampled individuals per genotype, *N*_*i*_, by the total number of individuals, *N*. We assume that parasite populations are much larger than host populations, and that they can thus be approximated with a deterministic description (i.e. without a sampling step).

### Matching allele models

The fitness matrices *w*^*H*^_*ij *_and *w*^*P*^_*ij *_can specify a broad range of types of host-parasite interactions. In particular, two important special cases can be specified: The matching allele (MA) model assumes that the parasite can infect the host only if it matches all interaction loci of the host. An infected host suffers a fitness cost of *s*_*H*_, while a non-infective parasite suffers a fitness cost of *s*_*P*_. For the fitness matrices, this implies that for a match at all alleles of genotypes *i *and *j*, *w*^*H*^_*ij *_= 1-*s*_*H *_and *w*^*P*^_*ij *_= 1; and else *w*^*H*^_*ij *_= 1 and *w*^*P*^_*ij *_= 1-*s*_*P*_. Unless stated otherwise we assume *s*_*H *_= *s*_*P *_= *s *(the effects discussed in this paper remain, however, qualitatively the same if one relaxes this assumption and assumes for example a strong selection on the parasite and variable selection on the host such as in [6,7]). The MA model is extreme in that the effect of an allele at an interaction locus depends very strongly on its genetic background (i.e. epistasis is very strong). At the other extreme of very weak epistasis is the multiplicative matching allele (MMA) model, which is specified as follows. The extreme matches (i.e. 0 or 2 matched loci) have the same fitness as in the MA. If the parasite matches only one interaction locus, the fitness values of the MMA are intermediate: *w*^*H*^_*ij *_= (1-*s*_*H*_)^1/2 ^and *w*^*P*^_*ij *_= (1-*s*_*P*_)^1/2^.

The MA and the MMA represent the extremes of very strong and no epistatic interactions. In order to study the RQD for a more general range of different models for the interaction between host and parasite, we consider the following set of fitness matrices: the fitness of the host-genotype *i *interacting with the parasite-genotype *j *depends only on the number of host loci matched by the parasite (*n*), i.e. *w*^*H*^_*ij *_= *w*^*H*^_*n *_= (1-*s*_*n*_); in addition, we set *w*^*H*^_0 _= 1, i.e. a host that is not recognized by the parasite at any interaction locus suffers from no fitness cost. By assumption host and parasite interact antagonistically, i.e. the higher the fitness of the parasite, the lower the fitness of the host. For simplicity, we assume that this antagonistic interaction takes the form *w*^*P*^_*n *_= *c/w*^*H*^_*n*_, i.e. the fitness of the parasite is inversely proportional to the fitness of the host (whereby the constant *c *is chosen such that *w*^*P*^_2 _= 1). For two loci, the system can thus be described by two parameters *s*_1 _and *s*_2_(the selection on a host matched at one or two loci, respectively). This generalized matching allele model (with inverse proportionality between host and parasite fitness) contains the MA model (*s*_1 _= 0, *s*_2 _*> *0) and the MMA model (*w*^*H*^_2 _= *w*^*H*^_1 _* *w*^*H*^_1_) but also a broad range of epistatic interactions of intermediate strength.

### Measuring selection on the modifier of recombination

We estimate the selection for larger or smaller recombination rates in the following way: Starting with randomly chosen initial genotype frequencies at interaction loci, we allow host and parasite populations to coevolve for 1,000 host generations, during which all hosts reproduce at a wildtype recombination rate *r*_*mm*_. After that, the mutant allele M is introduced in 50% of the host population, and after another 1,000 host generations, the frequency of M is recorded. The frequency change of the modifier, averaged over a large number of samples, is then used as a proxy for the selection on the modifier of recombination. We have verified this proxy using a simple one-locus/two-allele model with fitness values 1 and 1+s (see supplementary figure S1, Additional file 1).

## Authors' contributions

All three authors contributed substantially to designing, performing, analyzing, and interpreting the simulations. All authors read and approved the final manuscript.

## Supplementary Material

Additional file 1 Supplementary material. Contains the supplementary tables and figures that are mentioned in this article. Click here for file
