# Supplementary material for: The Red Queen and the persistence of linkage-disequilibrium oscillations in finite and infinite populations
Source: BMC Evol Biol. 2007 Nov 6;7:211. doi: 10.1186/1471-2148-7-211 (PMC2198919; doi:10.1186/1471-2148-7-211)
Supplement: Additional file 1 — Supplementary material. Contains the supplementary tables and figures that are mentioned in this article. [file 1471-2148-7-211-S1.pdf]

SUPPLEMENTARY MATERIAL

FIGURE LEGENDS

Figure S1

Relation between strength of selection, s, and frequency change of locus A (Δf_A) in a one locus two allele model (alleles *a* and *A*). Shown are simulation results for a deterministic model (dashed line) and a stochastic model with various population sizes (see legend). Allele *a* has fitness 1, allele *A* has fitness 1+*s*. Initially, f_A is set to 0.5 and after 1000 generations, the new frequency is measured. The figure shows that Δf_A is a good proxy for the strength of selection on the recombination modifier (as used in the main text): Δf_A corresponds to comparable selection coefficients in both a deterministic and a stochastic model.

Figure S2

Time course of the deterministic MMA with parameters $s_1 = 0.1$, $s_2 = 2\,s_1 - s_1^2$, $r = 0$, and $n_{pg} = 1$. Full black lines correspond to the frequency of genotype 00, full green lines to 11, dashed black lines to 01, and dashed green lines to 10. The initial allele frequencies are 1/2 at both loci in all three plots. The three plots describe the dynamics of the system for different initial host LD (parasites are initialized in linkage equilibrium): **(A)** no initial LD (i.e. all genotype frequencies are 1/4); **(B)** initial LD = 0.001; **(C)** initial LD = -0.001. Note that with a small positive or negative initial LD the system goes into a steady state with almost maximal positive or negative LD respectively. The lines were slightly shifted to enhance visibility.

FIGURES

Figure S1

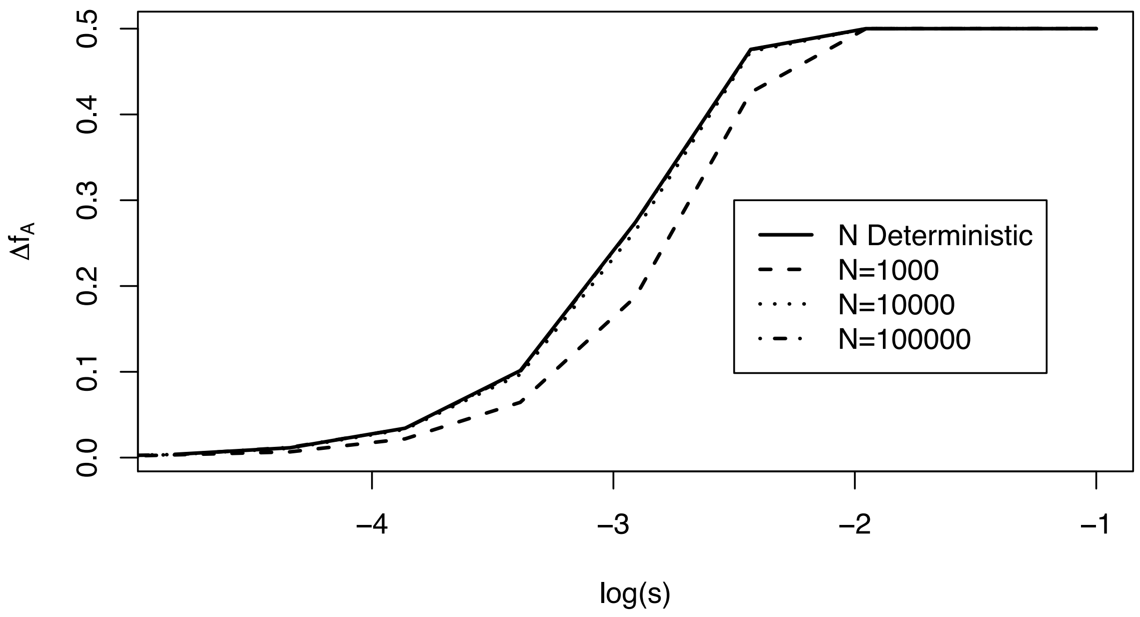

Figure S2

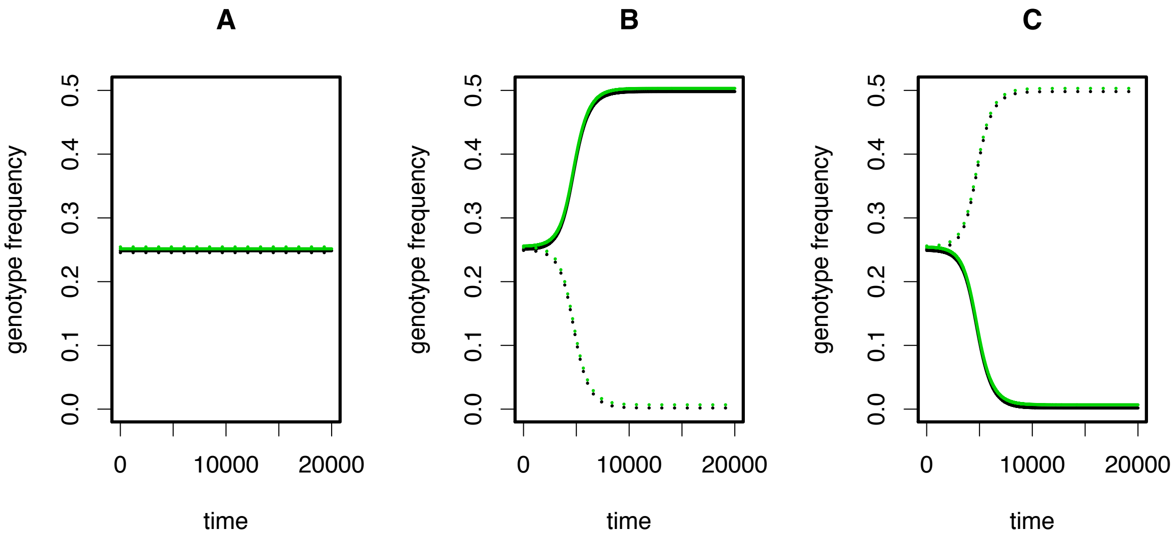

TABLES

Table S1 Maximum difference of linkage disequilibrium (LD) between host generations 10,000 and 20,000 as a proxy for long-term LD amplitudes, averaged over 1000 simulation runs with random initial conditions.

$s_H = s_P = 0.05$ (deterministic model)

	**r**									
**μ**	*0.0005*	*0.001*	*0.002*	*0.004*	*0.008*	*0.016*	*0.032*	*0.064*	*0.128*	*0.256*
10^{-7}	0.13726	0.07734	0.02960	0.01739	0.01588	0.01366	0.00766	0.00297	0.00330	0.00020
$2 * 10^{-7}$	0.13246	0.07272	0.02965	0.01660	0.01578	0.01344	0.00766	0.00289	0.00274	0.00019
$4 * 10^{-7}$	0.12213	0.07063	0.02794	0.01651	0.01526	0.01299	0.00761	0.00288	0.00280	0.00022
$8 * 10^{-7}$	0.11177	0.06278	0.02557	0.01582	0.01509	0.01286	0.00734	0.00263	0.00264	0.00012
$1.6 * 10^{-6}$	0.09793	0.05431	0.02245	0.01376	0.01377	0.01217	0.00669	0.00249	0.00210	0.00016
$3.2 * 10^{-6}$	0.07154	0.03932	0.01725	0.01162	0.01145	0.00963	0.00595	0.00222	0.00187	0.00007
$6.4 * 10^{-6}$	0.04476	0.02344	0.01018	0.00745	0.00692	0.00626	0.00434	0.00168	0.00143	0.00005
$1.3 * 10^{-5}$	0.01908	0.00983	0.00430	0.00321	0.00323	0.00269	0.00214	0.00100	0.00079	0.00004
$2.6 * 10^{-5}$	0.00370	0.00172	0.00082	0.00073	0.00074	0.00069	0.00054	0.00031	0.00021	0.00002
$5.1 * 10^{-5}$	0.00014	0.00006	0.00004	0.00004	0.00004	0.00003	0.00003	0.00002	0.00001	0.00000

$s_H = s_P = 0.05$ (stochastic model, N = 10 000)

	**r**									
**μ**	*0.0005*	*0.001*	*0.002*	*0.004*	*0.008*	*0.016*	*0.032*	*0.064*	*0.128*	*0.256*
10^{-7}	0.39285	0.38237	0.35501	0.30872	0.24434	0.18533	0.14370	0.11467	0.08400	0.05991
$2 * 10^{-7}$	0.38610	0.37495	0.34927	0.30603	0.24322	0.18294	0.14316	0.11262	0.08353	0.05983
$4 * 10^{-7}$	0.37647	0.36332	0.34162	0.29847	0.24268	0.18428	0.14418	0.11195	0.08320	0.05936
$8 * 10^{-7}$	0.36497	0.35377	0.33220	0.29560	0.24040	0.18281	0.14249	0.11205	0.08257	0.05859
$1.6 * 10^{-6}$	0.35100	0.34071	0.32314	0.28774	0.23696	0.18101	0.13954	0.11002	0.08155	0.05783
$3.2 * 10^{-6}$	0.33643	0.32798	0.30925	0.27731	0.23100	0.17741	0.13834	0.10745	0.07951	0.05668
$6.4 * 10^{-6}$	0.31613	0.30862	0.29419	0.26495	0.22209	0.17221	0.13494	0.10437	0.07719	0.05527
$1.3 * 10^{-5}$	0.29412	0.28591	0.27433	0.24860	0.21117	0.16505	0.12952	0.10165	0.07483	0.05275
$2.6 * 10^{-5}$	0.26469	0.25900	0.24731	0.22603	0.19408	0.15534	0.12166	0.09536	0.07078	0.04961
$5.1 * 10^{-5}$	0.23121	0.22415	0.21429	0.19902	0.17272	0.14089	0.11209	0.08789	0.06566	0.04675

$s_H = s_P = 0.5$ (deterministic model)

μ	r: 0.0005	0.001	0.002	0.004	0.008	0.016	0.032	0.064	0.128	0.256
10^{-7}	0.24909	0.19528	0.10645	0.04197	0.01951	0.01064	0.00523	0.00354	0.00213	0.00111
$2 * 10^{-7}$	0.24407	0.19270	0.10347	0.04240	0.02256	0.01131	0.00515	0.00352	0.00211	0.00112
$4 * 10^{-7}$	0.24253	0.19008	0.10411	0.03808	0.01873	0.01165	0.00522	0.00347	0.00210	0.00112
$8 * 10^{-7}$	0.22717	0.18297	0.09471	0.03714	0.01848	0.01163	0.00527	0.00357	0.00229	0.00152
$1.6 * 10^{-6}$	0.21200	0.16564	0.08699	0.03047	0.01774	0.01043	0.00474	0.00327	0.00216	0.00282
$3.2 * 10^{-6}$	0.19082	0.13332	0.06350	0.02801	0.01419	0.00765	0.00401	0.00274	0.00173	0.00188
$6.4 * 10^{-6}$	0.13508	0.09658	0.03721	0.01631	0.01170	0.00529	0.00299	0.00206	0.00124	0.00071
$1.3 * 10^{-5}$	0.06669	0.03489	0.01353	0.00973	0.00562	0.00224	0.00155	0.00103	0.00063	0.00037
$2.6 * 10^{-5}$	0.00672	0.00406	0.00167	0.00094	0.00066	0.00052	0.00040	0.00028	0.00018	0.00011
$5.1 * 10^{-5}$	0.00022	0.00009	0.00004	0.00004	0.00003	0.00003	0.00002	0.00002	0.00001	0.00001

$s_H = s_P = 0.5$ (stochastic model, N = 10 000)

μ	r: 0.0005	0.001	0.002	0.004	0.008	0.016	0.032	0.064	0.128	0.256
10^{-7}	0.38587	0.38124	0.37188	0.34826	0.29508	0.23808	0.18615	0.13704	0.09872	0.06936
$2 * 10^{-7}$	0.37652	0.37278	0.36403	0.34182	0.29480	0.23834	0.18572	0.13746	0.09859	0.06905
$4 * 10^{-7}$	0.36755	0.36358	0.35571	0.33506	0.29232	0.23811	0.18421	0.13571	0.09863	0.06958
$8 * 10^{-7}$	0.35789	0.35281	0.34525	0.32769	0.28980	0.23823	0.18356	0.13658	0.09855	0.06944
$1.6 * 10^{-6}$	0.34452	0.34147	0.33438	0.31870	0.28430	0.23608	0.18594	0.13655	0.09803	0.06940
$3.2 * 10^{-6}$	0.33018	0.32745	0.32080	0.30780	0.27906	0.23404	0.18314	0.13568	0.09839	0.06914
$6.4 * 10^{-6}$	0.31500	0.31222	0.30662	0.29463	0.26955	0.22896	0.18161	0.13461	0.09735	0.06858
$1.3 * 10^{-5}$	0.29724	0.29455	0.28909	0.28014	0.25792	0.22220	0.17673	0.13308	0.09613	0.06823
$2.6 * 10^{-5}$	0.27530	0.27344	0.26847	0.25944	0.24162	0.20956	0.16938	0.12918	0.09400	0.06664
$5.1 * 10^{-5}$	0.24897	0.24691	0.24184	0.23343	0.21768	0.19179	0.15709	0.12312	0.09017	0.06414

$s_H = 0.05$, $s_P = 0.9$ (deterministic model)

μ	r 0.0005	0.001	0.002	0.004	0.008	0.016	0.032	0.064	0.128	0.256
10^{-7}	0.03833	0.02479	0.01021	0.00299	0.00191	0.00139	0.00108	0.00091	0.00057	0.00014
$2 * 10^{-7}$	0.03456	0.02251	0.00941	0.00284	0.00182	0.00138	0.00108	0.00090	0.00057	0.00024
$4 * 10^{-7}$	0.03054	0.01993	0.00866	0.00272	0.00173	0.00135	0.00105	0.00092	0.00067	0.00038
$8 * 10^{-7}$	0.02624	0.01709	0.00752	0.00249	0.00169	0.00144	0.00116	0.00100	0.00127	0.00476
$1.6 * 10^{-6}$	0.02146	0.01409	0.00607	0.00212	0.00150	0.00120	0.00094	0.00085	0.00078	0.00101
$3.2 * 10^{-6}$	0.01616	0.01028	0.00442	0.00168	0.00128	0.00104	0.00080	0.00068	0.00050	0.00039
$6.4 * 10^{-6}$	0.01009	0.00623	0.00240	0.00117	0.00095	0.00077	0.00058	0.00048	0.00036	0.00022
$1.3 * 10^{-5}$	0.00433	0.00229	0.00090	0.00055	0.00048	0.00039	0.00031	0.00023	0.00016	0.00009
$2.6 * 10^{-5}$	0.00083	0.00042	0.00015	0.00012	0.00012	0.00010	0.00008	0.00006	0.00004	0.00003
$5.1 * 10^{-5}$	0.00004	0.00002	0.00001	0.00001	0.00001	0.00001	0.00001	0.00000	0.00000	0.00000

$s_H = 0.05$, $s_P = 0.9$ (stochastic model, N = 10 000)

μ	r 0.0005	0.001	0.002	0.004	0.008	0.016	0.032	0.064	0.128	0.256
10^{-7}	0.11464	0.11451	0.11394	0.11298	0.11096	0.10705	0.10079	0.08934	0.07363	0.05621
$2 * 10^{-7}$	0.11214	0.11168	0.11113	0.11041	0.10826	0.10505	0.09853	0.08757	0.07264	0.05583
$4 * 10^{-7}$	0.10920	0.10926	0.10857	0.10750	0.10580	0.10247	0.09642	0.08593	0.07163	0.05529
$8 * 10^{-7}$	0.10590	0.10613	0.10553	0.10484	0.10319	0.09997	0.09425	0.08421	0.07044	0.05469
$1.6 * 10^{-6}$	0.10287	0.10252	0.10259	0.10175	0.10021	0.09732	0.09188	0.08259	0.06933	0.05417
$3.2 * 10^{-6}$	0.09986	0.09979	0.09934	0.09859	0.09731	0.09409	0.08931	0.08067	0.06808	0.05347
$6.4 * 10^{-6}$	0.09611	0.09612	0.09600	0.09493	0.09383	0.09125	0.08638	0.07838	0.06649	0.05269
$1.3 * 10^{-5}$	0.09276	0.09252	0.09213	0.09183	0.09014	0.08796	0.08376	0.07616	0.06472	0.05165
$2.6 * 10^{-5}$	0.08848	0.08807	0.08826	0.08757	0.08624	0.08449	0.08039	0.07349	0.06267	0.05054
$5.1 * 10^{-5}$	0.08427	0.08395	0.08382	0.08301	0.08243	0.08044	0.07675	0.07061	0.06073	0.04928
